# Poly(lactic acid)/Carbon Nanotube Fibers as Novel Platforms for Glucose Biosensors

**DOI:** 10.3390/bios2010070

**Published:** 2012-02-27

**Authors:** Juliano Elvis Oliveira, Luiz Henrique Capparelli Mattoso, Eliton Souto Medeiros, Valtencir Zucolotto

**Affiliations:** 1 PPGCEM, Departamento de Engenharia de Materiais (DEMA), Universidade Federal de São Carlos (UFSCAR), Rodovia Washington Luis, km 235, Monjolinho, 13.565-905, São Carlos, SP, Brazil; E-Mail: julianoufmg@yahoo.com.br; 2 Laboratório Nacional de Nanotecnologia para o Agronegócio (LNNA), Embrapa Instrumentação (CNPDIA), Rua XV de Novembro, 1452, Centro, 13.560, 970 São Carlos, SP, Brazil; E-Mail: mattoso@cnpdia.embrapa.br; 3 Departamento de Engenharia de Materiais (DEMAT), Universidade Federal da Paraíba (UFPB), Cidade Universitária, 58.051-900, João Pessoa, PB, Brazil; E-Mail: eliton_s@yahoo.com; 4 Laboratório de Nanomedicina e Nanotoxicologia (LNN), Instituto de Física de São Carlos, Universidade de São Paulo, 13.560-970, P.O. Box 369, São Carlos, SP, Brazil

**Keywords:** nanofibers, glucose biosensor, carbon nanotube, poly(lactic acid)

## Abstract

The focus of this paper is the development and investigation of properties of new nanostructured architecture for biosensors applications. Highly porous nanocomposite fibers were developed for use as active materials in biosensors. The nanocomposites comprised poly(lactic acid)(PLA)/multi-walled carbon nanotube (MWCNT) fibers obtained via solution-blow spinning onto indium tin oxide (ITO) electrodes. The electrocatalytic properties of nanocomposite-modified ITO electrodes were investigated toward hydrogen peroxide (H_2_O_2_) detection. We investigated the effect of carbon nanotube concentration and the time deposition of fibers on the sensors properties, viz*.*, sensitivity and limit of detection. Cyclic voltammetry experiments revealed that the nanocomposite-modified electrodes displayed enhanced activity in the electrochemical reduction of H_2_O_2_, which offers a number of attractive features to be explored in development of an amperometric biosensor. Glucose oxidase (GOD) was further immobilized by drop coating on an optimized ITO electrode covered by poly(lactic acid)/carbon nanotube nanofibrous mats. The optimum biosensor response was linear up to 800 mM of glucose with a sensitivity of 358 nA·mM^−1^ and a Michaelis-Menten constant (K_M_) of 4.3 mM. These results demonstrate that the solution blow spun nanocomposite fibers have great potential for application as amperometric biosensors due to their high surface to volume ratio, high porosity and permeability of the substrate. The latter features may significantly enhance the field of glucose biosensors.

## 1. Introduction

Glucose biosensors constitute a very important and widespread class of enzymatic biosensors, due to the relevance of glucose determinations in biomedical diagnosis and food technology [1,2,3,4]. Glucose oxidase (GOD) from *Aspergillus niger* is a slightly elongated globular flavoprotein with an average diameter of 8 nm that catalyzes the oxidation of β-D-glucose to D-glucono-δ-lactone and H_2_O_2_ using molecular oxygen as an electron acceptor [5,6,7]. This reaction occurs via a redox process, in which the reduction process comprises the oxidation of β-D-glucose to D-glucono-δ-lactone, which is then hydrolyzed to gluconic acid. Following, the flavine adenine dinucucleotide (FAD) is reduced to FADH2 [5,6]. In the oxidative process, the reduced GOD is reoxidized by oxygen and H_2_O_2_ obtained.

Different electrodes design, immobilization approaches and materials for enzyme support have been investigated aiming at improving the efficiency of glucose detection [8,9,10,11,12]. Among these new investigated architectures, polymer nanofibers have attracted much attention because of their unique properties such as enzyme supports and high surface area [13,14,15,16,17,18]. These nanostructures can be generated by various methods of fiber spinning [19,20,21,22] including electrospinning and solution blow spinning. The latter method seems to be the simplest one, through which fibers with small diameters and a very fast production rate can be obtained [21].

Recent papers [10,18,23,24] reported that nanofibers are excellent supports for protein immobilization due to a variety of polymers that can be spun, high porosity and the interconnectivity of mats and the nanofibers surface, which can be modified to benefit enzyme immobilization. These unique features ensure the application of electrospun and solution blow spun nanofibers in biosensors [17] and biocatalysis [25]. To improve the electrochemical properties of biosensors, several authors [26,27,28,29] have indicated the use of nanocomposites based on polymer nanofibers and carbon nanotubes as enzyme supports. 

Multi-walled carbon nanotubes have been used in electroanalysis as a component of nanocomposites due their mechanical strength, large aspect ratio, high electronic properties and easy functionalization [30,31,32]. Multi-walled carbon nanotubes (MWCNTs) consist of several concentric cylinders of graphitic shells with a layer spacing of 0.3–0.4 nm. MWCNTs are considered a mesoscale graphite system, whereas the single walled carbon nanotubes (SWCNTs) are a single large molecule [30]. Due to their structure, MWCNTs provide many active sites that can enhance the sensitivity of electrochemical biosensors. Moreover, the open ends of MWCNTs increase electron transfer rate similar to graphite edge-plane electrodes, while SWCNTs have a very slow electron transfer rate and low specific capacitance, similar to the graphite basal plane [12]. These are some of the advantages of multi-walled carbon nanotubes in electrochemical sensor and biosensor applications due their unique structure and properties such as high surface area and conductivity and absorbability and fast electron transfer rate.

Poly(lactic acid) fibers have been successfully obtained by electrospinning [33,34] and solution blow spinning [21] from a variety of solvents for applications in biosensors, biomaterials and filtration. A recent study [14] reported that biotin has been successfully incorporated into PLA nanofibers through electrospinning and used as a biosensor. Manesh *et al*. developed an electrospun based nanofibrous composite of poly(methyl methacrylate) and MWCNTs, to be used in the detection of glucose [15]. The study confirmed that fibrous morphologies resulted in an excellent matrix to immobilization of glucose oxidase. 

The production and characterization of poly(lactic acid)/carbon nanotube composites obtained by the solution blow spinning process has been previously described by our group [35]. In the present study we show the use of solution blow spun mats for the fabrication of modified electrodes for glucose biosensors. The spun fiber modified electrodes were characterized using cyclic voltammetry and used as modified electrodes for glucose biosensing via amperometric measurements toward detection of hydrogen peroxide.

## 2. Experimental Section

### 2.1. Materials

Multi-walled carbon nanotubes, glutaraldehyde (GA) 25% in H_2_O, glucose oxidase (GOD) from *Aspergillus niger* (type II, 24,800 units/g) and D-glucose were purchased from Sigma Aldrich. Poly(lactic acid) (Mw = 75,000 g·mol^−1^) was supplied by Biomater (São Carlos, Brazil). All other agents used in the experiments were of analytical grade and were purchased from Synth (São Paulo, Brazil). Deionized water was obtained from a Milli-Q system (Millipore). Indium-doped tin oxide (ITO)-coated glass plates of 10 mm × 5 mm area (specific surface resistance of 8–12 Ω) were obtained from Delta Technologies (Minnesota, USA) and used as electrodes for the amperometric sensors. Before each experiment, ITO plates were rinsed with isopropyl alcohol and washed with distilled water.

### 2.2. Methods

Solution blow spun mats were fabricated using methodology described elsewhere [35]. Typically, PLA and MWCNT were dissolved in chloroform/acetone mixture (3:1 v/v). Spinning of the composite solutions was performed at a feed rate of 120 μL/min with an air pressure of 0.4 MPa. A distance of 12 cm was kept between the syringe tip and collector. The length of the protruding inner nozzle was 2 mm, and the ratio diameter between the concentric nozzles was 0.5. These parameters were kept constant for all experiments. We investigated the effects of both concentration of carbon nanotubes (0–3 wt%) and deposition time (1–15 min) on PLA fibers deposited over ITO-coated glass plates. The area of the working electrode was 5 mm × 5 mm.

Then, electrochemical experiments were performed using a three electrode system with an Ag/AgCl reference electrode, a 1 cm^2^ platinum foil counter electrode and a modified ITO electrode was used as the working electrode. The bipotentiostat used was a µSTAT200 (DropSens, Ouviedo, Spain). Modified electrodes were characterized by cyclic voltammetry using H_2_SO_4_ (0.1 M) and [Fe(CN)_6_]^3−/4−^ (5 mM) and compared with the results of the ITO electrode. Chronoamperograms were taken for the electrocatalytic studies using a solution of 0.2 M of hydrogen peroxide. All measurements were carried out in 0.1 M phosphate buffer at 25 °C. The effects of carbon nanotubes concentration and time deposition on the sensitivity and limit of detection (LOD) of hydrogen peroxide sensors had been quantitatively investigated. Sensitivity was calculated as the slope of calibration curve. Limit of detection [36] of sensors and biosensors could be calculated according to the Equation (1):

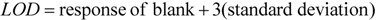
(1)

After that, for biosensor assays, PLA membranes were spun onto ITO electrodes for 1 min from a solution of 6 wt% PLA–1 wt% MWCNT. Different amounts of GOD (0.25–2.0 U/μL) and GA (0–2.5% v/v) were dissolved in phosphate buffer solution pH 7.4, NaCl (0.1 M) for optimization of immobilization. GOD was immobilized on the spun fibers by a drop coating procedure. Briefly, 10 μL of an enzyme solution (0.25–2.0 U/μL) was dropped into the modified electrode, allowing immobilization via cross linking using 5 μL of glutaraldehyde solutions in different concentrations (0–2.5% v/v). The effect of glutaraldehyde solution concentration (used for enzyme immobilization) on the morphology of fibers was investigated by a DSM960 Zeiss scanning electron microscopy (SEM), after gold-coating with a sputter coater (Balzers, SCD 050). For descriptive statistic, fiber diameters were measured with the aid of image software (Image J, National Institutes of Health, USA). Average fiber diameter and distribution were determined from about 100 random measurements using micrographs representative of fiber morphology. The sensitivity and LOD of several biosensors were studied by chronoamperometry at the working potential of 0.45 V *vs*. Ag/AgCl electrode to detect hydrogen peroxide produced in the enzyme reaction. A glucose solution (0.2 M) was added gradually into the phosphate buffer (0.1 M) at several pH (5.5–9) and 25 °C. 

Finally, the kinetic parameters of the immobilized glucose oxidase, *I*_max_ and *K_M_*, were determined according to Equation (2):

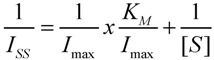
(2)
where *I_SS_* is the steady-state current after the addition of substrate, and S is the bulk concentration of substrate. *I*_max_, is the maximum current measured under saturated substrate solution and reflects the intrinsic characteristics of the enzyme. K_M_ indicates the substrate concentration at which the reaction rate is half of *I*_max_. 

## 3. Results and Discussion

### 3.1. Electrode Characterization

**Figure 1:**
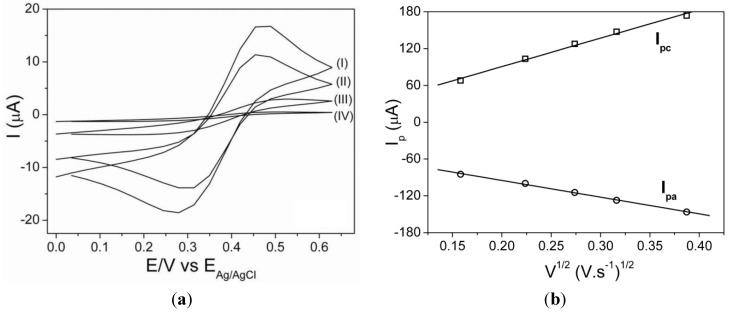
(**a**) Effect of time deposition of poly(lactic acid)(PLA) fibers on cyclic voltammetric response at a scan rate of 50 mV·s^−1^. (I) 0 min; (II) 1 min; (III) 3 min and (IV) 15 min deposition time; (**b**) Peak current *versus* scan rate for mats deposited for 1 min on modified indium tin oxide (ITO) electrodes.

Cyclic voltammetry was performed in a 5 mM [Fe(CN)_6_]^3−/4−^ probe solution containing H_2_SO_4_ (0.1 M). As shown in Figure 1(a), a pair of well-defined redox peaks of [Fe(CN)_6_]^3−/4−^ probe was observed for the ITO bare electrode (Figure 1(a-I)), as expected. These peaks were still well-defined for electrodes containing fibers deposited for 1 min (Figure 1(a-II)), indicating the porosity and permeability of the spun mat on the ITO surface. A remarkable decrease of peak current was obtained at 3 min (Figure 1(a-III)) and 15 min (Figure 1(a-IV)) of deposition, suggesting that the bulkier fibers blocked the electron exchange between the redox probe and electrode surface.

Electron transfer kinetics in nanocomposite fiber modified ITO electrodes was monitored using cyclic voltammetry (CV). Figure 1 shows typical voltammograms obtained from the ITO electrode modified by PLA-MWCNT fibers in H_2_SO_4_ (0.1 M) and K_3_[Fe(CN)_6_] (5 mM) solution. A pair of well-defined, quasi-reversible redox peaks is observed for the nanocomposite fiber modified ITO electrode. The anodic peak potential (E_pa_) and cathodic peak potential (E_pc_) are close to 489 and 279 mV, respectively, representing the redox couple characteristic of Fe(CN)_6_^4−^/Fe(CN)_6_^3−^ in potassium ferricyanide. The anodic and cathodic potential peaks positions shifted to positive and negative directions upon increasing the scan rates, respectively. The peak current showed linear dependence with square root of scan rate in the range of 25–150 mV/s, indicating the occurrence of surface confined process at the electrode. The formal potential (E_o_) defined as the average of E_pa_ and E_pc_ is 384 mV, is in agreement with the reported values in the literature [37]. The peak-to-peak separation (ΔE_p_) was 210 mV at a scan rate of 100 mV/s, which is smaller than 248 mV for ITO electrodes [38] and 380 mV for ITO electrodes modified with multi-walled carbon nanotubes/polyaniline bilayers [27] obtained at the same scan rate. This indicated a more reversible direct electron transfer achieved between nanocomposite fibers and electrode.

Standard heterogeneous electron transfer rate constant (k_s_) for the Fe(CN)_6_^3−/4−^ redox couple between nanocomposite fibers and the ITO surface was estimated at 7.2 s^−1^ by the Laviron method [39]. The latter value is higher than that of carbon nanotube powder microelectrodes (2.48 s^−1^) [40], or carbon nanotube-based electrodes (1.53 ± 0.45 s^−1^) [41]. These results suggest that the non-woven fiber mat greatly facilitates the electron transfer kinetics in the multi-walled carbon nanotubes on ITO electrodes. This is attributed to the high porosity and permeability of nanocomposite fiber mat deposited on the ITO surface.

### 3.2. Influence of Carbon Nanotubes Concentration

**Figure 2:**
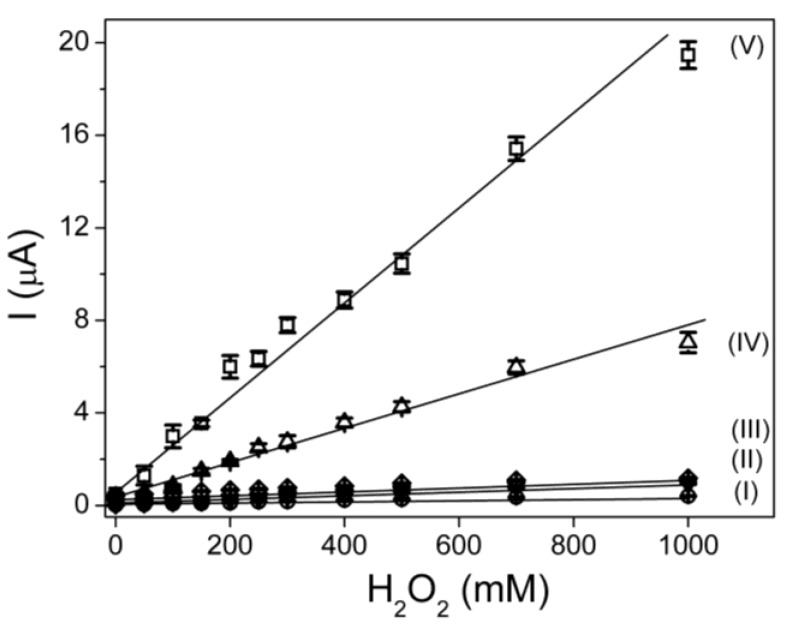
Calibration curve from chronoamperometry data using the modified electrodes containing different amounts of MWCNTs: (I) 0 wt%; (II) 0.1 wt%; (III) 0.5 wt%; (IV) 3 wt% and (V) 1 wt%.

The effect of MWNT content on sensitivity of hydrogen peroxide electrode was investigated in a range of 0 and 3 wt% of MWCNTs. The calibration plot under the experimental conditions between 0 and 3 wt% is shown in Figure 2. 

**Table 1:** Effect of experimental variables on the limit of detection of sensors.

MWCNT (%)	LOD (mM)	Sensitivity (nA·mM^−1^)
0	8.3 ± 0.4	15 ± 2
0.1	1.01 ±0.3	5 ± 3
0.5	0.4 ±0.3	22 ± 3
1	0.3 ± 0.1	213 ± 4
3	1.4 ± 0.3	83 ± 3
**Time of deposition (min)**	**LOD (mM)**	**Sensitivity (nA·mM^−1^)**
1	0.2 ± 0.1	208 ± 4
3	1.2 ±0.1	36 ± 7
15	1.5 ±0.2	2 ± 8

Repeatability and reproducibility are standardized terms used by literature [36,37], and they are associated with the precision of measurements obtained by the same material using the same methodology and analysis conditions. Repeatability is the variability of the measurements obtained by the same person using the same methodology, equipment and analysis conditions. Reproducibility is the variability of the measurement system caused by differences in operator behavior or equipment. The reproducibility study for the PLA-MWCNT 1 wt% sensor was carried out by using 3 sensors for each time of deposition of fibers. Each sensor was used only once. An increase in sensitivity and a decrease in the limit of detection (Table 1) were observed upon increasing MWNT content, reaching a maximum (sensitivity) and minimum (limit of detection) at 1 wt%. Further increase in the amount of MWNTs led to a decrease in sensitivity and an increase in the limit of detection, possibly because of fiber morphology effect as will be discussed in Section 3.4. As a result, 1 wt% MWNTs was used for preparation of the glucose biosensors.

### 3.3. Influence of Deposition Time on Sensor Properties

**Figure 3:**
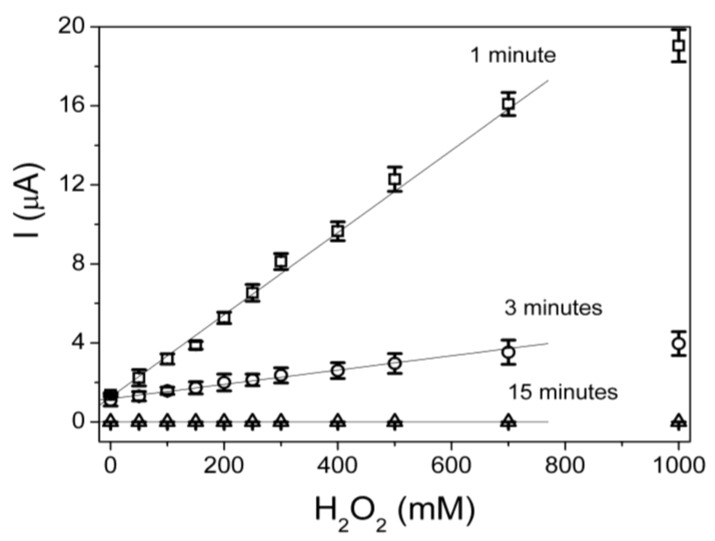
Calibration curve from chronoamperometry data using the modified electrodes prepared using different fiber deposition times.

Figure 3 shows the influence of the density of nanocomposite fibers on the sensor properties. The density of fibers can be controlled by controlling parameters including deposition time, polymer concentration and rate of injection in the blow spinning process [21]. All measurements were performed in triplicate using different sensors (reproducibility). Figure 3 shows the calibration plots under the experimental conditions between 1 and 15 min of time of deposition. It can be observed that when the time of deposition was 1 min, the fiber modified electrodes show the highest sensitivity and lowest limit of detection (Table 1) toward hydrogen peroxide detection.

The average limit of detection and its standard deviation as a function of the experimental variables (carbon nanotube content and time of fiber deposition) are listed in Table 1.

### 3.4. Enzyme Immobilization

**Figure 4:**
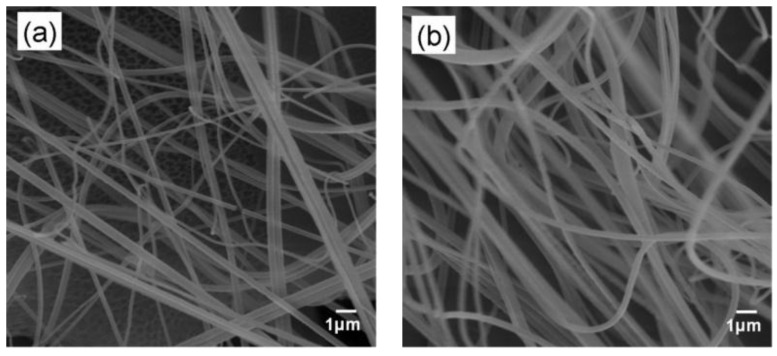
SEM images of the PLA-1%MWCNT fibers (**a**) before immobilization and (**b**) after immobilization of GOD using 0.125% GA.

Glucose oxidase was immobilized on the spun fibers by a drop coating procedure. SEM analyses revealed that the immobilization of glucose oxidase in composite mats proceeded without considerable changes in the diameter and the morphology of the fibers, which were homogeneous, smooth and non-porous (Figure 4). The average diameter of the PLA/MWCNT fibers was 247 ± 120 nm. After enzyme immobilization by drop coating, the average fiber diameters were close to that of the nanocomposite fibers 312 ± 97 (0% GA), 387 ± 153 (0.125% GA), 385 ± 184 (0.25% GA), 305 ± 87 (1.25% GA) and 494 ± 213 nm (2.5% GA).

### 3.5. Amperometric Determination of Glucose at Poly(lactic acid)/Carbon Nanotubes Modified Electrodes

The average limit of detection and its standard deviation as a function of the experimental variables (glutaraldehyde, enzyme concentration and pH) are listed in Table 2. 

**Table 2:** Effect of experimental variables in limit of detection of biosensors.

Glutaraldehyde (% v/v)	LOD (mM)	Sensitivity (nA·mM^−1^)
0	7.6 ± 0.8	31 ± 1
0.125	2.8 ± 0.2	144 ± 4
0.25	1.2 ± 0.3	140 ± 5
1.25	3.1 ± 0.4	15 ± 1
2.5	5.5 ± 0.4	6 ± 1
**Enzyme Concentration(U·µL^−1^)**	**LOD (mM)**	**Sensitivity (nA·mM^−1^)**
0.25	2.5 ± 0.4	7 ± 2
0.5	2.3 ± 0.5	23 ± 4
0.75	1.6 ± 0.3	358 ± 9
1	1.4 ± 0.3	140 ± 5
2	3.8 ± 0.4	26 ± 3
**pH**	**LOD (mM)**	**Sensitivity (nA·mM^−1^)**
5.4	3.5 ± 0.3	10 ± 4
6	2.3 ± 0.4	22 ± 1
7	1.5 ± 0.2	145 ± 6
8	1.5 ± 0.1	204 ± 6
9	1.4 ± 0.2	147 ± 3

All measurements were performed in triplicate using different biosensors (reproducibility). This result shows the effect of the concentration of glutaraldehyde enzyme casting solution on the biosensor response. In the range of 0–0.25% (v/v) glutaraldehyde, the biosensor response increased upon on increasing glutaraldehyde concentration. However, excess of glutaraldehyde in the range of 0.25–2.5% (v/v) led to a sudden decrease in the biosensor response. Amino groups of glucose oxidase can react with glutaraldehyde. Since the glucose biosensor is based on the production of hydrogen peroxide, its increase in solution strongly depends on the enzymatic catalysis of glucose oxidation, and any change in enzyme activity would affect biosensor sensitivity. The immobilized glucose oxidase on electrode surface would become detached easily if glutaraldehyde had not been used. However, higher glutaraldehyde concentration would denature most of the enzymes and lead to a decrease in sensitivity. Therefore, the maximum response was observed when the concentration of glutaraldehyde used for enzyme immobilization was 0.25% (v/v).

Calibration plots of glucose biosensors with different enzyme loading (0.25–2.0 U/μL) were determined at an applied potential of +0.45 V. The biosensor sensitivity increased and the limit of detection decreased upon increasing enzyme loading, reaching a maximum at 0.75 U/μL, as shown in Table 2. Further increasing the amount of glucose oxidase led to a decrease in sensor response, possibly because of saturation and blocking of active sites on the enzyme in the electrode.

The effect of pH on glucose biosensor calibration is shown in Table 2. Changes in pH are one of the most important parameters capable of altering glucose oxidase activities in aqueous solution. The effect of pH on the sensitivity and limit of detection of biosensor electrodes was investigated within a pH range of 5.4–8.9 at room temperature. Sensitivity and limit of detection as a function of pH are shown in Table 2. The optimum pH of immobilized glucose oxidase in nanocomposite modified electrodes was found at *ca.* 8. It is known, however, that the optimum pH of glucose oxidase varies from 5.0 to 7.0. Therefore, a shift in the optimum pH towards more basic values was observed when glucose oxidase was immobilized into the nanocomposite fiber matrix. The three dimensional structure of the active site of the enzyme may be affected by the immobilization procedure. These glucose biosensors were more sensitive to pH changes in acidic media than alkaline.

### 3.6. Kinetic Parameters of the Immobilized Glucose Oxidase on the Nanocomposite Fibers Modified Electrodes

**Figure 5:**
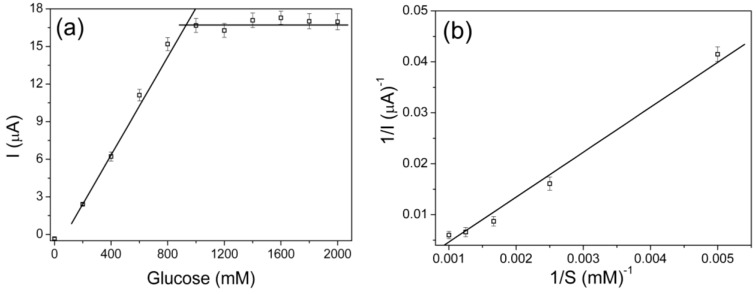
Calibration curve built up from chronoamperometry data using the optimum biosensors (**a**) and Lineweaver-Burk plot (**b**).

Kinetic parameters of PLA–MWCNT/ITO glucose oxidase biosensor at +450 Mv (*vs*. Ag/AgCl), and pH 8.9 for the successive additions of glucose were studied by chronoamperometry and are shown in Figure 5. All measurements were performed in triplicate using different biosensors (reproducibility).

The sensitivity (ΔE_p_) was 488 ± 8 nA·mM^−1^ and the limit of detection (LOD) was 1.7 ± 0.3 mM in the linear range of 2–800 mM using the optimized biosensor. In these electrodes, the enzyme was immobilized at pH 8.0, GA concentration of 0.25% v/v and enzyme concentration of 0.75 U/μL. The current response became constant for a glucose concentration beyond 900 mM (R = 0.99). This fact indicates a Michaelis-Menten kinetic mechanism for the enzyme catalyzed process [42]. From Lineweaver-Burk plot the K_M_ value for the immobilized glucose oxidase was estimated at 4.3 mM. This increase in K_M_ can be due to the conformational changes of the glucose oxidase which indicates that the immobilization method used allowed high accessibility of the substrate to its active sites caused by the decreased diffusion limitations. When glucose oxidase is immobilized on the surface of fibrous nanocomposite mats, their high surface area and porosity enable active sites exposed to glucose oxidase to interact more easily with the substrate [42,43]. The same applies for electrospun nanofibrous membrane [15], conducting polymers [44] and TiO_2_ nanoparticles [45] as it has been recently reported.

## 4. Conclusions

We developed a solution blow spun fibrous nanocomposite (PLA/MWCNT) modified electrode for detection of hydrogen peroxide. Electron transfer kinetics in fiber modified-ITO electrodes was monitored using cyclic voltammetry. The effects of fiber deposition time and carbon nanotubes concentration on the sensitivity and limit of detection was investigated and optimized via electrochemical reduction of H_2_O_2_. We found that 1 min of fiber deposition and an addition of 1 wt% carbon nanotubes into PLA fibers are the optimum conditions for the hydrogen peroxide sensors. Solution blow spun fibers/glucose oxidase biosensors showed a sensitivity of 358 nA·mM^−1^ and detection limit of 0.08 mM for glucose, which are among the best reported values in the literature. The biosensor also exhibited a wide linear range (0–900 μ·mM) which follows typical Michaelis-Menten saturation kinetics. The K_M_ value for the optimum immobilized glucose oxidase was determined to be 4.3 mM. These features demonstrate that the solution blow spun nanocomposite fibers can significantly improve the biosensor properties and offer great potential for applications as amperometric biosensors.
